# Clinical Characteristics of Hospitalized Neonates With Hypofibrinogenemia: A Retrospective Cohort Study

**DOI:** 10.3389/fped.2020.00589

**Published:** 2020-09-22

**Authors:** Weijun Zhou, Yichun He, Qin Li, Ying Li, Yongchun Su, Li Yan

**Affiliations:** Department of Pediatrics, Chongqing Yubei District People's Hospital, Chongqing, China

**Keywords:** neonate, fibrinogen deficiency, hemorrhage, thrombocytopenia, blood coagulation factor deficiencies

## Abstract

**Background:** Neonatal hypofibrinogenemia is often asymptomatic but can manifest as hemorrhage.

**Objective:** This study was conducted to characterize clinical characteristics of neonates with hypofibrinogenemia and identify factors associated with hemorrhage.

**Methods:** This was a retrospective study of neonates with plasma fibrinogen level (FIB) ≤1.0 g/L who were hospitalized at the Neonatology Department, People's Hospital, Chongqing, China, from January 2012 to December 2017. Based on severity, patients were grouped into severe, moderate, and mild hypofibrinogenemia (FIB < 0.5 g/L, 0.5 g/L ≤ FIB < 0.7 g/L, and 0.7 g/L ≤ FIB ≤ 1.0 g/L, respectively). Clinical characteristics associated with hemorrhage were analyzed.

**Results:** Among 330 neonates, 52.7% showed mild hypofibrinogenemia, 25.5% had moderate hypofibrinogenemia, and 21.8% had severe hypofibrinogenemia. Severe hypofibrinogenemia was not associated with gestational age, but the mild form was frequent in neonates with low/normal birthweight (*P* = 0.018). Approximately 80.6% of neonates presented hypofibrinogenemia as variable combinations of thrombocytopenia or coagulopathies. Hemorrhage occurred in 38.8% of the cases, 60.9% of which were mild. Hemorrhage manifested as puncture site bleeding (47.7%) or spontaneous skin/mucous membrane bleeding (34.2%). The degree of hypofibrinogenemia was not associated with the severity or occurrence of hemorrhage. Among patients with hypofibrinogenemia and bleeding, 53.4% of the cases with coagulopathies showed mild hemorrhage, 85.7% of the cases with thrombocytopenia had moderate bleeding, while 53.8% of the cases with coagulopathy and thrombocytopenia showed severe hemorrhage.

**Conclusion:** Neonatal hypofibrinogenemia is often comorbid and occurs with thrombocytopenia and/or coagulopathies. Although hemorrhage is not associated with the degree of hypofibrinogenemia, it may be severe when hypofibrinogenemia co-occurs with coagulopathies and/or thrombocytopenia.

## Introduction

Fibrinogen (coagulation factor I, FIB)is a 340 kDa hexameric protein composed of alpha, beta, and gamma chains (1). Fibrinogen is synthesized by the liver (2) and circulates in the blood as one of the most abundant plasma proteins, with a normal concentration range of 1.5–3.5 g/dl (3). This glycoprotein is an important component of the coagulation system involved in hemostasis (4). Following injury to tissue and blood vessels, fibrinogen is cleaved by thrombin to fibrin, which leads to the formation of a blood clot composed of cross-linked fibrin and incorporated blood cells (including platelets, erythrocytes, and white blood cells) (5).

Fibrinogen disorders can be categorized into afibrinogenemia (complete absence of fibrinogen), hypofibrinogenemia (low plasma levels of fibrinogen), and dysfibrinogenemia (normal fibrinogen levels (1.5–3.5 g/L) with low functional activity) (6). The most common causes of hypofibrinogenemia include reduced synthesis (especially in liver diseases), increased consumption of clotting factors (e.g., in patients with cancer or sepsis with disseminated intravascular coagulation (DIC), or during treatments with tissue plasminogen activator), and hemodilution (e.g., during massive transfusion) (7). Congenital hypofibrinogenemia is secondary to a variety of inherited gene defects (8). Although individuals with afibrinogenemia present with umbilical cord bleeding, mucosal bleeding, and intracerebral and/or intraabdominal bleeding (9–11), hypofibrinogenemia is often asymptomatic and presents during traumatic bleeding or invasive surgical procedures (3). Treatment with fibrinogen concentrate (fibrinogen replacement therapy) is the most suitable therapy when fibrinogen levels fall below 0.5 g/L (3).

Neonates are highly susceptible to hypofibrinogenemia due to physiologic immaturity and, moreover, because fibrinogen cannot readily cross transplacentally from the mother to the fetus. Although numerous studies have focused on the pathogenesis of hypofibrinogenemia or afibrinogenemia in neonates, the vast majority of them were case reports with only a small number of patients (10, 12–24). However, only a few reports examining the clinical features of neonatal hypofibrinogenemia in large cohorts have been published till date. Therefore, in view of the above and due to a paucity of literature on this subject, in this study, we aimed to characterize the clinical characteristics of neonates with hypofibrinogenemia admitted to the Neonatology Department, People's Hospital, Chongqing, China, and identify the clinical factors associated with hemorrhage.

## Materials and Methods

### Patients

This was a retrospective study of neonates with a confirmed diagnosis of low plasma fibrinogen level (FIB ≤ 1.0 g/L) who were hospitalized at the Neonatology Department of People's Hospital in the Chongqing Yubei District, Chongqing, China, between January 1, 2012 and December 31, 2017. The inclusion criteria were as follows: (1) patient age 28–42 weeks and (2) low plasma fibrinogen level (FIB ≤ 1.0 g/L) detected within 1 month after birth during at least one of three measurements. The exclusion criteria were as follows: (1) patients with severe cardiopulmonary disease and (2) patients who received invasive ventilation. This study was approved by the Ethics Committee of Chongqing Yubei District People's Hospital, Chongqing, China. The need for informed consent for the analyses was waived off by the committee as the study was retrospective and data were deidentified.

### Fibrinogen Plasma Levels

Fibrinogen was measured during routine workup in all patients included in the study in order to confirm the presence of coagulation disorders or acute infection before surgery or diagnostic interventions. Only the pre-treatment and pre-interventional fibrinogen values were used for statistical analyses. For routine fibrinogen analyses, blood samples were derived from peripheral venous punctures on the day of admission to the hospital. Fibrinogen levels were measured according to the Clauss method as previously described (25).

### Study Groups

In this study, based on the degree of severity of FIB, the patient cohort was grouped into three: severe hypofibrinogenemia (FIB < 0.5 g/L), moderate hypofibrinogenemia (0.5 g/L ≤ FIB < 0.7 g/L), and mild hypofibrinogenemia (0.7 g/L ≤ FIB ≤ 1.0 g/L).

### Therapy

Neonates with FIB < 0.5 g/L were infused with fresh frozen plasma or cryoprecipitate. Symptomatic treatments were given to patients who showed a progressive increase in subcutaneous bleeding and those who had severe infections, fever, and other clinical conditions.

### Follow-Up and Clinical Data

For each patient, data pertaining to hospital admission and discharge were extracted from our medical health records. The data collected included sex, gestational age at birth, birthweight, FIB level, site, and severity of hemorrhagic event (if any), presence of thrombocytopenia and/or any other coagulation disorder, and treatment regimen. Mild hemorrhage was defined as any bleeding event with no accompanying anemia, moderate hemorrhage was defined as any bleeding event accompanied by anemia that did not reach severe hemorrhage levels, and severe hemorrhage was defined as any bleeding event accompanied by either a decline in hemoglobin levels by ≥30 g/L or symptomatic intracranial hemorrhage. A coagulation disorder was confirmed by an activated partial thromboplastin time (APTT) of ≥60 s and a prothrombin time (PT) ≥20 s. Thrombocytopenia was confirmed when platelet counts were <100 × 10^9^/L.

The study was followed up thrice based on the electronic records of the hospital discharge database. All the groups of patients were followed up for an average of 3.6 years (range 0.1–6.5 years) until death or truncation (end of the observation period on June 31, 2019), whichever came first. Mortalities were recorded in the database. The observation period was the entire interval for which data are available for a patient between the time of their discharge and either death or truncation.

### Statistical Analyses

All data were analyzed by using SPSS 18.0 (SPSS Inc., Chicago, IL, USA). Continuous variables with a normal distribution are represented as mean ± standard deviation, and categorical variables are shown as numbers and percentages. Comparisons between groups were carried out by using Student's *t*-test and χ^2^-tests, as appropriate. *P* < 0.05 was considered statistically significant.

## Results

### Baseline Characteristics

Among 330 neonates included in the analyses, 186 (60.3%) were male and 144 (39.7%) were female. The patient cohort was stratified into four subgroups based on gestational age: patients <34 months, patients aged 34–36 months, patients aged 37–41 months, and patients ≥42 months. Delivery was full term (37–41^+6^ weeks' gestation) for 194 neonates (58.8%) and pre-term (<37 weeks' gestation) for 120 neonates (36.4%), with 8 infants born at <32 weeks' gestation, 24 infants born at 32–33^+6^ weeks' gestation, and 88 infants born at 34–36^+6^ weeks' gestation. We had further stratified the patient cohort into two subgroups based on birthweight, namely, patients with birthweight ≥2.5 kg (normal) and those having birthweight between 1.5 and 2.5 kg (low). There were 98 cases (29.7%) with low birthweight, 214 cases (64.8%) with normal birthweight, and 18 cases (5.5%) with fetal macrosomia.

### Comparison of Gestational Age at Birth and Birthweight Among the Study Groups

Based on the degree of severity of hypofibrinogenemia, we grouped the neonatal cohort (*n* = 330) into mild hypofibrinogenemia (*n* = 174, 52.7%), moderate hypofibrinogenemia (*n* = 84, 25.5%), and severe hypofibrinogenemia (*n* = 72, 21.8%). Although there were no significant differences in gestational age among the groups (*P* = 0.061), a statistically significant difference in birthweight was observed among the three patient groups (*P* = 0.018). Notably, mild hypofibrinogenemia in infants was found to be associated with low or normal birthweight (Table 1).

**Table 1:** Comparison of gestational age at birth, birthweight, incidence of hemorrhage, degree of hypofibrinogenemia, and its association with comorbidities among the study groups.

	**Mild hypofibrinogenemia (*n* = 174)**	**Moderate hypofibrinogenemia (*n* = 84)**	**Severe hypofibrinogenemia (*n* = 72)**	***P***
Gestational age, *n* (%)				0.061
<34 months	14 (43.8)	10 (31.3)	8 (25.0)	
34–36^+6^ months	48 (54.5)	22 (25.0)	18 (20.5)	
37–41^+6^ months	104 (53.6)	46 (23.7)	44 (22.7)	
≥42 months	8 (50.0)	6 (37.5)	2 (12.5)	
Birthweight, *n* (%)				0.018
1.5–2.5 kg	50 (51.0)	28 (28.6)	20 (20.4)	
≥2.5 kg	124 (53.4)	56 (24.1)	52 (22.4)	
With hemorrhage, *n* (%)	116 (67.7)	44 (52.4)	42 (58.3)	0.075
Degree of hemorrhage, *n* (%)				0.142
Mild	42 (65.6)	22 (55.0)	14 (58.3)	
Moderate	20 (31.3)	16 (40.0)	6 (25.0)	
Severe	2 (3.1)	2 (5.0)	4 (16.7)	
Comorbidities, *n* (%)				0.006
Hypofibrinogenemia alone	6 (38.5)	4 (30.8)	2 (30.8)	
Hypofibrinogenemia + coagulopathy^***^	58 (53.4)	28 (11.7)	2 (35.0)	
Hypofibrinogenemia + thrombocytopenia	0 (0)	4 (85.7)	2 (14.3)	
Hypofibrinogenemia + coagulopathy + thrombocytopenia^**^	14 (38.5)	6 (7.7)	2 (53.8)	

** *P < 0.05*,

*** *P < 0.001 vs. the hypofibrinogenemia + thrombocytopenia group*.

### Comorbidities

Among 330 neonates with hypofibrinogenemia, about 80.6% (*n* = 266) presented with abnormal coagulation or thrombocytopenia, about 35.8% (*n* = 118) had abnormal liver function, about 10.6% (*n* = 38) had infection, about 29.7% (*n* = 98) had experienced asphyxia or hypoxia, about 7.9% (*n* = 26) showed severe hyperbilirubinemia, about 4.2% (*n* = 14) had scleroderma, and about 0.6% (*n* = 2) presented with DIC.

### Hemorrhagic Events

Hemorrhagic events occurred in 128 neonates (38.8%). Out of these, 78 cases (47.0%) showed mild bleeding, 42 cases (16.1%) had moderate hemorrhage, and 8 cases (36.9%) had severe hemorrhage. Among the infants who experienced a bleeding event, the most common manifestation was hemorrhage at a puncture site that required a prolonged compression time (*n* = 61, 47.7%) followed by spontaneous skin/mucosal hemorrhage (*n* = 44, 34.2%), gastrointestinal hemorrhage (*n* = 26, 20.3%), intracranial hemorrhage (*n* = 10, 7.8%), hemorrhage at the umbilical cord stump (*n* = 10, 7.8%), and subconjunctival hemorrhage (*n* = 2, 1.6%). Among 26 neonates with gastrointestinal hemorrhage, 10 cases had visible bloody vomit or bloody stool or black stool, 10 cases were identified during routine stool examinations by the presence of erythrocytes or a positive fecal occult blood test, and 6 cases were confirmed during invasive procedures such as gastric lavage or gastrointestinal drainage. Patients (*n* = 10) with intracranial hemorrhage who were diagnosed by cranial magnetic resonance imaging did not show any specific clinical manifestation. However, hemorrhage was not seen in the respiratory tract, urinary tract, liver, muscle, joint, or spleen of any patient.

### Factors Associated With the Incidence of Hemorrhage

In this study, there were no significant differences in the incidence of hemorrhage among the mild, moderate, and severe hypofibrinogenemia groups (Table 1). Although the presence of thrombocytopenia as a comorbidity was not found to be associated with the incidence of hemorrhage, the presence of hypofibrinogenemia in combination with coagulopathies and/or thrombocytopenia was significantly associated with the occurrence of hemorrhage (*P* = 0.038 and *P* = 0.005, respectively) (Table 2).

**Table 2:** Associations between the presence of hypofibrinogenemia in combination with or without coagulopathies and/or thrombocytopenia as comorbidities and the incidence of hemorrhage.

**Disorder**	**Without hemorrhage**		**With hemorrhage**		**Statistical values**
	**No. of cases**	**Percentage**	**No. of cases**	**Percentage**	
Hypofibrinogenemia alone	38	76.0	12	24.0	χ^2^ = 7.953, *P* = 0.047
Hypofibrinogenemia + coagulopathy^*^	134	60.3	88	39.7	
Hypofibrinogenemia + thrombocytopenia	10	62.5	6	37.5	
Hypofibrinogenemia + coagulopathy + thrombocytopenia^**^	20	47.6	22	52.4	

* *P < 0.05*,

** *P < 0.01 vs. the hypofibrinogenemia-alone group*.

### Factors Associated With the Severity of Hemorrhage

In neonates who experienced a bleeding event, the severity of the hemorrhage did not differ significantly among the mild, moderate, and severe hypofibrinogenemia groups (Table 3). However, the degree of hemorrhage did vary significantly among the groups depending on whether hypofibrinogenemia occurred in combination with coagulopathies and/or thrombocytopenia (Table 2). Mild hemorrhage was more likely to occur when hypofibrinogenemia co-occurred with other indicators of coagulation abnormalities, moderate hemorrhage was observed when hypofibrinogenemia concomitantly occurred with thrombocytopenia, whereas severe hemorrhage was found to be more common in patients with both coagulation dysfunction and thrombocytopenia (*P* = 0.006) (Table 2).

**Table 3:** Associations between the degree of hypofibrinogenemia and the severity of hemorrhage.

**Disorder**	**Mild hemorrhage**		**Moderate hemorrhage**		**Severe hemorrhage**		**Statistical values**
	**No. of cases**	**Percentage**	**No. of cases**	**Percentage**	**No. of cases**	**Percentage**	
Mild hypofibrinogenemia	42	65.6	20	31.3	2	3.1	χ^2^ = 6.879, *P* = 0.142
Moderate hypofibrinogenemia	22	55.0	16	40.0	2	5.0	
Severe hypofibrinogenemia	14	58.3	6	25.0	4	16.7	

### Changes in Fibrinogen During Follow-Up

Among 330 neonates with hypofibrinogenemia, about 63.0% of the cases (*n* = 208) were not administered with fresh frozen plasma or cryoprecipitate infusion. However, out of these 208 cases, about 32.7% (*n* = 68) of the cases were followed up to evaluate the response to therapy. Notably, in these 68 neonates who were not administered with fibrinogen substitutes, there was no significant difference in FIB levels before and after follow-up (0.75 ± 0.19 and 0.85 ± 0.36 g/L, respectively; *t* = −2.011; *P* = 0.06). On the contrary, about 37.0% of the remaining cases (*n* = 122) were infused with fresh frozen plasma or cryoprecipitate, out of which about 77.0% (*n* = 94) of the cases were followed up for changes in FIB levels after therapy. Further, in these 94 cases, FIB levels were found to be significantly higher after treatment with fresh frozen plasma or cryoprecipitate than those observed prior to therapy (1.18 ± 0.49 and 0.61 ± 0.28 g/L, respectively; *t* = −8.157; *P* = 0.001).

## Discussion

This retrospective cohort provides novel insights into the clinical characteristics of hypofibrinogenemia in neonates and further reveals comorbid coagulopathies and/or thrombocytopenia to be predictive factors that may influence the risk of hemorrhage.

Fibrinogen is mainly involved in the final stages of blood coagulation process, and therefore, the absence of fibrinogen can lead to coagulation dysfunction and hemorrhage. Nevertheless, neonatal hypofibrinogenemia often manifests without hemorrhage or only with slight hemorrhage (3, 12). Consistent with the above findings, we found that only 38.8% of neonates showed hemorrhage, which mostly presented as the mild form in approximately 60.9% of the cases. Similar to previous studies, hemorrhage in neonates manifested as puncture site bleeding, spontaneous skin/mucous membrane bleeding, and umbilical cord bleeding (9, 15, 17, 22, 23, 26). Nonetheless, hypofibrinogenemia can also present with potentially life-threatening hemorrhage such as intracranial bleeding (10, 14, 16, 19, 20), which was also detected in a small number of infants in our study.

Consistent with other reported findings, in our analyses, approximately 52.7% of the neonates showed mild hypofibrinogenemia, where none of the patients had a low FIB value of <0.1 g/L (27). Additionally, 25.5% of the patients had moderate hypofibrinogenemia, and approximately 21.8% of them presented the severe form.

About 80.6% of the neonates presented hypofibrinogenemia as variable combinations of coagulopathies and/or thrombocytopenia. This could be attributed to the effects of various stress factors on an immature liver, which in turn result in low levels of coagulation factors in patients with hypofibrinogenemia. Indeed, 35.8% of the neonates had an abnormal liver function, and approximately 29.7% of them experienced asphyxia or hypoxia, which was associated with hepatic dysfunction (28). Therefore, taken together, the possibility of occurrence of hypofibrinogenemia needs to be considered in neonates with liver function damage or a previous history of hypoxia/ischemia.

Due to the plausible role of hepatic immaturity in the pathogenesis of hypofibrinogenemia observed in the neonates of our study, we further investigated whether the degree of hypofibrinogenemia was associated with gestational age at birth. Notably, we found no significant correlations between the degree of hypofibrinogenemia and gestational age among the patients, which is in complete agreement with a previous report on FIB (29). Interestingly, we observed that infants with either low or normal birthweight displayed mild hypofibrinogenemia, in contrast to those with fetal macrosomia, where moderate hypofibrinogenemia was more often presented. Of relevance to this, a previous study suggested that gestational diabetes-induced fetal macrosomia was associated with the downregulation of the alpha-chain of fibrinogen (30). Collectively, these findings raise the possibility that hospitalized neonates with macrosomia may be at an increased risk of hypofibrinogenemia.

Another important finding of this study was that, in most of the cases, neonatal hypofibrinogenemia concomitantly occurred with other coagulation abnormalities and/or thrombocytopenia, which suggests that physiologic immaturity and hepatic dysfunction are the primary causes of hypofibrinogenemia. This finding highlights the importance of screening for other coagulation abnormalities when hypofibrinogenemia is detected.

Although the incidence of hemorrhage was not associated with FIB, as reported previously (17), the incidence of bleeding events was significantly associated with the presence of other coagulation abnormalities. Moreover, the incidence of hemorrhage was not significantly different when hypofibrinogenemia occurred alone or in combination with thrombocytopenia. This could be attributed to the small sample of the latter, and hence, the study may have been underpowered to detect a significant difference. Nonetheless, the incidence of hemorrhage in patients with hypofibrinogenemia and thrombocytopenia (37.5%) was visibly greater than that observed in patients with hypofibrinogenemia alone (24.0%) and in patients with hypofibrinogenemia and coagulopathies (39.7%). Therefore, these findings indicate that the identification of other coagulation abnormalities and/or thrombocytopenia in neonates with hypofibrinogenemia needs to be interpreted as a predisposed risk for bleeding.

It is widely accepted that fibrinogen replacement therapy should be instigated in neonates with FIB < 0.5 g/L. Nonetheless, the difference in the mean FIB values (prior to follow-up) of patients administered with fibrinogen substitutes (0.61 ± 0.28 g/L) and of those who were not treated (0.75 ± 0.19 g/L) suggests that there may have been differing clinical perspectives regarding intervention during follow-up. Expectedly, in contrast to a small and a non-significant elevation of FIB levels observed in untreated neonates after follow-up, the administration of fresh frozen plasma or cryoprecipitate significantly elevated FIB levels in those treated, indicating that fibrinogen replacement therapy is effective in elevating FIB in neonates with hypofibrinogenemia.

This study has some limitations. As this was a retrospective study, the results may have been prone to selection bias or information bias. Further, the single-center study design may limit the generalizability of the findings in this study. In addition, the small sample size stratified into a few subgroups may have underpowered our study, and thus, detection of real differences between subgroups may have been further compromised. Moreover, the possibility of the presence of unknown confounding factors cannot be ruled out in our analyses. Furthermore, the impact of intervention on the incidence of hemorrhage was not evaluated.

In conclusion, most cases of neonatal hypofibrinogenemia were associated with only mild hemorrhage, although severe bleeding events did occur in a small number of cases. Neonatal hypofibrinogenemia is a comorbid disorder and often occurs concomitantly with thrombocytopenia and/or other coagulopathies. Although hemorrhage was not associated with the degree of hypofibrinogenemia, it is a predisposing factor in patients with hypofibrinogenemia in combination with coagulopathies and/or thrombocytopenia. Our study emphasizes the importance of screening for coagulation abnormalities or thrombocytopenia, if neonatal hypofibrinogenemia is detected.

## Data Availability Statement

The raw data supporting the conclusions of this article will be made available by the authors, without undue reservation, to any qualified researcher.

## Ethics Statement

The studies involving human participants were reviewed and approved. This study was approved by the ethics committee of Chongqing Yubei District People's Hospital, and the retrospective study was exempted from informed consent. Written informed consent to participate in this study was provided by the participants' legal guardian/next of kin. Written informed consent was obtained from the minor(s)' legal guardian/next of kin for the publication of any potentially identifiable images or data included in this article.

## Author Contributions

WZ drafted the manuscript. LY was involved in the design and implementation of the study. YH, QL, and YL were involved in data collection and study population. YS performed literature searches and took responsibility for the integrity of the data and the accuracy of the data analysis. All authors read and approved the final manuscript.

## Conflict of Interest

The authors declare that the research was conducted in the absence of any commercial or financial relationships that could be construed as a potential conflict of interest.
